# Cholelithiasis, Gut Microbiota and Bile Acids after Bariatric Surgery—Can Cholelithiasis Be Prevented by Modulating the Microbiota? A Literature Review

**DOI:** 10.3390/nu16152551

**Published:** 2024-08-03

**Authors:** Natalia Komorniak, Jan Pawlus, Katarzyna Gaweł, Viktoria Hawryłkowicz, Ewa Stachowska

**Affiliations:** 1 Department of Human Nutrition and Metabolomics, Pomeranian Medical University in Szczecin, 71-460 Szczecin, Poland; viktoria.hawrylkowicz@pum.edu.pl (V.H.); ewa.stachowska@pum.edu.pl (E.S.); 2 Department of General Mini-Invasive and Gastroenterological Surgery, Pomeranian Medical University in Szczecin, 71-460 Szczecin, Poland; pawlus.jan@gmail.com; 3 Department of Gastroenterology, Pomeranian Medical University in Szczecin, 71-460 Szczecin, Poland; ka.gawel88@gmail.com

**Keywords:** bariatric surgery, microbiota, cholelithiasis, bile acids, gallstone, obesity, ursodeoxycholic acid

## Abstract

Background: Cholelithiasis is one of the more common complications following bariatric surgery. This may be related to the rapid weight loss during this period, although the exact mechanism of gallstone formation after bariatric surgery has not been fully elucidated. Methods: The present literature review focuses on risk factors, prevention options and the impact of the gut microbiota on the development of gallbladder stones after bariatric surgery. Results: A potential risk factor for the development of cholelithiasis after bariatric surgery may be changes in the composition of the intestinal microbiota and bile acids. One of the bile acids—ursodeoxycholic acid—is considered to reduce the concentration of mucin proteins and thus contribute to reducing the formation of cholesterol crystals in patients with cholelithiasis. Additionally, it reduces the risk of both asymptomatic and symptomatic gallstones after bariatric surgery. Patients who developed gallstones after bariatric surgery had a higher abundance of *Ruminococcus gnavus* and those who did not develop cholelithiasis had a higher abundance of *Lactobacillaceae* and *Enterobacteriaceae*. Conclusion: The exact mechanism of gallstone formation after bariatric surgery has not yet been clarified. Research suggests that the intestinal microbiota and bile acids may have an important role in this.

## 1. Introduction

Gallbladder stones are one of the most common conditions of the biliary system. They are estimated to affect 10% of men and 20% of women in the United States of America [1]. They are also the most common cause of planned surgery, estimated at 700,000 cases a year [2]. In Poland, the percentage of patients with gallbladder stones is 20% [3]. A number of factors are believed to influence the development of this disease. They can be divided into modifiable and those whose modification is not possible. Non-modifiable factors include age, gender, ethnic and genetic factors. On the other hand, factors that can be influenced by the patient include, among others, hypertriglyceridemia, a high-calorie diet, obesity, and sudden weight reduction [4].

It is worth noting that the risk factors for gallbladder stones in the general population may differ from those in patients who have undergone bariatric surgery [5,6,7]. In this group of patients in particular, rapid weight loss appears to be an important element in the development of gallbladder stones. Indeed, it was found that after bariatric surgery, there was a significant increase in the percentage of patients diagnosed with gallbladder stones from 21.6% to 52.4%. This increase was mainly observed in a group of patients undergoing malabsorptive surgery (Roux-en-Y gastric bypass) [1]. Studies indicate that the use of ursodeoxycholic acid significantly reduces the incidence of gallbladder stones after bariatric surgery. Moreover, symptomatic gallbladder stones are found in only 36% of patients diagnosed with gallbladder stones postoperatively [8].

Studies show that obese patients experience changes in the composition of the intestinal microbiota (dysbiosis), and that bariatric surgery affects both microbiota and bile acid composition [9,10]. In addition, there are some studies indicating that the gastrointestinal microbiota may promote the development of gallstones by regulating bile acid metabolism and related signaling pathways [11].

Considering the increasing prevalence of obesity and the associated increase in the number of bariatric surgeries, it is essential to understand the risk factors for gallstone formation in this particular population. Identifying these factors can help plan effective strategies to prevent gallstones after surgery. This review aims to explore risk factors, prevention options and the role of the gut microbiota in the development of gallstones after bariatric surgery.

By examining the interplay between microbial modification of bile acids and the development of gallbladder stones, we hope to contribute to the development of a protocol for management and preventive care measures in this special group of patients.

## 2. Methods

The present literature review focuses on risk factors, prevention options and the impact of the gut microbiota on the development of gallbladder stones after bariatric surgery. A literature review was conducted based on the PubMed Database. The keywords were checked and combined for the following terms: bariatric surgery, sleeve gastrectomy, Roux-en-Y gastric bypass, laparoscopic gastric banding, cholelithiasis, gallstones, bile acids, microbiota and ursodeoxycholic acid. Studies that were not in the English language, letters to editors and abstracts to conferences were excluded.

## 3. Historical Overview of Bariatric Surgery

Obesity was declared a global epidemic by the World Health Organization in 1997. The disease is estimated to affect 650 million people worldwide [12]. Various methods and combinations of these methods are used to treat obesity. Modification of diet and introduction of physical exercise can be distinguished in conservative treatment. Pharmacological treatment (including treatment with GLP-1 analogs) is also used. In addition to conservative treatment, the most radical option is a surgical treatment called bariatric surgery [13].

Indications for surgery are determined by BMI. The basic eligibility criterion for bariatric surgery until 2022 was a BMI of 40 kg/m^2^. In 2022, after the publication of new recommendations, this threshold was lowered to 35 kg/m^2^ without comorbidities. If type 2 diabetes is present, the lowered threshold is 30 kg/m^2^ [14]. Polish guidelines, however, still set the eligibility threshold for bariatric surgery at a BMI of 40 kg/m^2^ as a stand-alone factor and 35 kg/m^2^ with comorbidities [15].

Bariatric surgeries are aimed at weight reduction. They can be divided into restrictive and malabsorptive (aimed at reducing nutrient absorption). The main representative of restrictive procedures is sleeve gastrectomy. It is currently the most popular and widely used type of bariatric surgery. It was originally described in 1988 as a modification of another bariatric surgery, the duodenal switch. In 1999, it was first performed laparoscopically and since 2000 it has been used as a separate procedure [16]. The procedure is relatively easy to perform by laparoscopic means. It involves cutting out most of the stomach and creating a sleeve from the remaining portion for the patient’s food intake. A 36F Bougie probe is used for this purpose. The ease of the operation is influenced by the lack of need for anastomosis [17]. Compared to another restrictive technique, the adjustable gastric band (LAGB), sleeve gastrectomy has been shown to reduce patient weight more effectively. This is thought to be related to a reduction in serum ghrelin levels [18]. Another well-established operation is Roux-en-Y gastric bypass (RYGB), or gastrointestinal exclusion by the Roux-en-Y method. The origins of this procedure date back to the 1960s and 1970s. So this operation has the longest history and observation [19]. This procedure involves the creation of a small gastric pouch. The preparation starts from the minor curvature. Then, after opening the net pouch, a transverse cut is made through the stomach. The incision line is guided in the direction of the angle of His, after previously inserting a 36F probe into the stomach. This results in a small gastric reservoir, which is then anastomosed to the intestinal loop. In our center, we perform a side-to-side anastomosis with a linear stapler. After the first anastomosis is performed, the small intestine is disconnected and a second intestinal–intestinal anastomosis is performed. The procedure is completed by closing the mesenteric defects in the Petersen space. The length of subsequent loops has been changed several times over time. However, it was found that a common loop length of less than 150 cm causes malnutrition [20]. A third commonly performed operation has become the mini gastric bypass (MGB or OAGB). This procedure also involves excluding a section of the small intestine from absorption. However, a single anastomosis is performed with the gastric reservoir, which in this case is longer. The anastomosis is performed around 200 cm distal to the ligament of Treitz. The MGB was originally used as a salvage procedure after a gunshot wound that required pyloric resection. It was later adapted for bariatric surgery [21]. The name OAGB refers to a modification of the original method, used by Miguel Carbajo, who linked the intestinal loop to the gastric pouch. This was intended to reduce the accumulation of bile in the stomach and esophagus. He also published the very good results of this method in weight reduction [22].

The three types of surgery discussed above comprise around 90 percent of the procedures performed worldwide. Recently, the United States of America has seen a significant increase in sleeve gastrectomies performed relative to RYGB. This has to do with the technical ease of the operation and comparable results [23].

## 4. Etiopathogenesis of Cholelithiasis in General Population

Cholelithiasis is the multifactorial disease and gallstones usually form because of the slow removal of bile from the gallbladder [24]. Bile mainly consists of water, bile acids, cholesterol, fatty acids, bilirubin, phospholipids, proteins and inorganic compounds. Under proper conditions, they are soluble in bile and then excreted into the gastrointestinal tract. However, disturbances in the compositions on bile affect the solubility of these substances. In this way, some of the bile remaining in the gallbladder can precipitate as a sludge or microliths and then turn into gallstones [24,25]. Motility disorders of the gallbladder, hepatic hypersecretion of cholesterol and its increased intestinal absorption, gut microbiota disorders, slow intestinal motility and the accumulation of mucin gel are also involved in this process [26,27]. What is more, research that has been carried out so far confirms the involvement of genetic factors in the pathogenesis of cholelithiasis. The mutations that have been identified so far are responsible for the abnormal secretion of bile acids and cholesterol and its transfer into the bile (ABCG5, ABCG8, CYP7A1, FXR and LDLR), disorders of cholesterol metabolism, gallbladder contractility (ADRB3), its emptying (CCKAR) and lipoprotein production (ApoE and ApoB-100) [28].

According to the composition, there are two major types of gallstones: cholesterol and pigment, which are also known as black stones. Cholesterol stones mainly form in the gallbladder because of increased bile lithogenicity, whereas pigment stones arise due to excessive secretion of bilirubin into the bile in diseases involving hemolysis and are usually present in bile ducts [24,25,26].

There are many risk factors of cholelithiasis and some of them can be modified [26]. Risk factors for cholesterol stones include the following: female sex, age ≥ 40 y, pregnancy, obesity, metabolic syndrome, family history, rapid weight loss (>1.5 kg/week), prolonged fasting, bariatric surgery, genes, diabetes mellitus type 2, drugs (such as estrogens, ceftriaxone, fibrates and thiazide diuretics), low physical activity, high-calorie diet and hypertriglyceridemia; meanwhile, factors for pigment stones are as follows: liver cirrhosis, Crohn’s disease, hemolytic anemia and long-term total parenteral nutrition [24,25,26,27,28].

## 5. Factors That Favor the Development of Cholelithiasis after Bariatric Surgery

Cholelithiasis is one of the more common complications following bariatric surgery (it is estimated that up to 30% of patients may be affected, which is five times higher than the healthy population) [29,30]. The majority (75%) of gallstones (GSs) form within the first two years after surgery [31,32]. This may be related to the rapid weight loss during this period, although the exact mechanism of GS formation after bariatric surgery has not been fully elucidated [32]. We know that lipolysis causes increased cholesterol excretion and mobilization, producing lithogenic bile; bypassing the duodenum (as part of some bariatric procedures) leads to less cholecystokinin secretion and thus less gallbladder motility [5]. Potentially rapid weight loss after surgery results in increased cholesterol saturation of bile, decreased bile acid secretion, increased mucin secretion (up to 10–20 times) and ultimately leads to decreased gallbladder emptying and thus bile stagnation [33,34]. A meta-analysis by Dai et al. [35] indicates that risk factors for gallbladder stone development after bariatric surgery include female gender and white race. In contrast, there was no statistical significance between the risk of developing gallbladder stones and smoking, RYGB surgery, preoperative BMI, the presence of hypertension, diabetes and dyslipidemia [35]. Interestingly, a meta-analysis by Amorim-Cruz et al. [30] indicates that female gender and mean preoperative BMI are not associated with an increased risk of developing symptomatic gallbladder stones. In addition, none of the assessed bariatric surgery types had a strong impact on GD risk; laparoscopic gastric banding (LAGB) was associated—with an 82% probability—with lower odds of de novo symptomatic GD [30]. In contrast, a meta-analysis by Wan et al. [36] focusing on the incidence of gallstones after RYGB and SG surgery showed that those undergoing SG had a 35% lower incidence of gallstones. In addition, those who underwent SG surgery were significantly less likely to undergo cholecystectomy than those who underwent RYGB. It is noteworthy that SG surgery was associated with a significantly lower incidence of cholelithiasis than RYGB surgery, only <2 years after surgery [36].

## 6. Microbial Modification of Bile Acids

**Figure 1:**
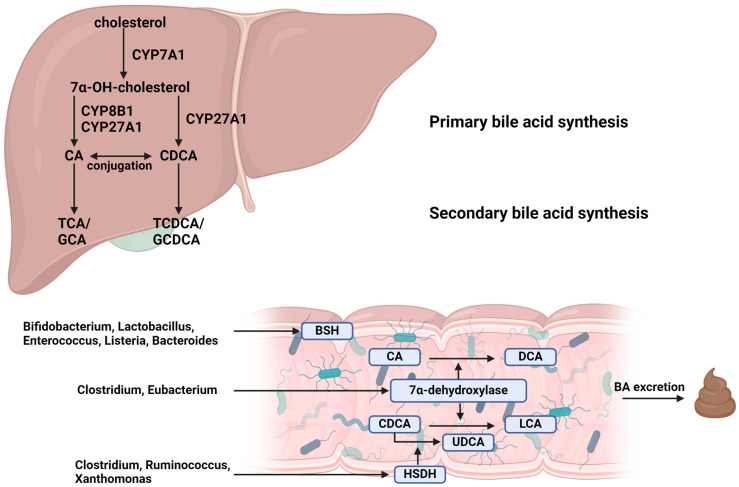
Synthesis of the bile acids and microbial transformation [37,38]. CA—cholic acid; CDCA—chenodeoxycholic acid; TCA—taurocholic acid; GCA—glycocholic acid; TCDCA—taurochenodeoxycholic acid; GCDCA—glycochenodeoxycholic acid; BSH—bile salt hydrolase; DCA—deoxycholic acid; LCA—lithocholic acid; UDCA—ursodeoxycholic acid; HSDH—hydroxysteroid dehydrogenase; BA—bile acids. Created with BioRender.com.

Primary bile acids are formed in hepatocytes; cholesterol is converted to 7α-OH-cholesterol by CYP7A1 or alternatively CYP27A1 (thus catalyzing further synthesis). In the presence or absence of CYP8A1, basic bile acids (BAs), cholic acid (CA) or chenodeoxycholic acid (CDCA), are synthesized (Figure 1) [37].

Primary BAs are conjugated with taurine/glycine, thereby increasing hydrophilicity, before being secreted in the bile. In the intestine, secondary BAs are converted from primary BAs by gut microbes. Bacteria with bile salt hydrolase (BSH) activity, including *Bifidobacterium*, *Lactobacillus*, *Clostridium* and *Enterococcus*, deconjugate primary conjugated BAs to taurine/glycine. Bacteria, including *Clostridium* and *Eubacterium*, which express 7α-dehydroxylase, convert CA to DCA and CDCA to LCA. Another enzyme, HSDH, expressed by bacteria including *Clostridium*, *Ruminococcus* and *Xanthomonas*, converts CDCA to UDCA. The first step in BA modification is deconjugation, in which the bacterial enzyme BSH removes amino acids, such as taurine or glycine, from conjugated bile acids, converting them to their free forms—many bacteria from the *Bacteroides*, *Firmicutes* and *Actinobacteria* clusters are responsible for this process [39]. The following step of converting primary fatty acids to secondary bile acids (cholic acid (CA) to deoxycholic acid (DCA) and chenodeoxycholic acid (CDCA) to lithocholic acid (LCA)) requires the enzyme 7α-dehydroxylase, which is expressed by bacteria of the *Lachnospiraceae* and *Peptostreptococcaceae* families [37,40]. The next stage of transformation is oxidation and epimerization. These processes are catalyzed by bacterial hydroxysteroid dehydrogenase (HSDH) enzymes, which change the structure of bile acids, making them less toxic and more water-soluble. These are bacteria from the phyla *Actinobacteria*, *Proteobacteria* and *Firmicutes* [41,42,43]. The activity of these bacteria in modifying bile acids is crucial for maintaining BA homeostasis and influencing liver function. These modified bile acids can serve as signaling molecules, influencing various processes and altering bile acid profiles that act on key metabolic receptors. The final step is desulfurization and reconjugation, carried out by bacteria which have the ability to remove sulfate groups from bile acids or reconjugate bile acids with other molecules such as amino acids. Bacteria involved in desulfurization are *Clostridium* species *Ruminococcus* and *Xanthomonas*. Reconjugation of unconjugated BAs with phenylalanine, tyrosine and leucine is an important part of the gut–hepatic axis. Bacteria involved in reconjugation are *Enterocloster*, *Enterococus* and *Bacteroides*, which are capable of reconjugating bile acids with amino acids such as phenylalanine, tyrosine and leucine, and *Bifidobacterium*, which can add various amino acids. The reconjugation process alters the solubility and function of bile acids, affecting their role in lipid digestion and absorption [44,45].

During the desulfurization process, bacterial enzymes called sulfatases remove sulfate groups from bile acids. This reduction in sulfate groups increases the rate at which bile acids are reabsorbed from the intestines into the bloodstream, affecting their overall circulation and impact on metabolism. After the deconjugation of bile acids (and their eventual modification by oxidation or reduction), some bacteria can recouple them with various amino acids. This alters not only their solubility, but also their interactions with bile acid receptors and their signaling capabilities [37].

It is worth remembering that the microbiota has the ability to influence bile acid signaling. Microbiota-modified bile acids can interact with host receptors, significantly affecting metabolic and immune pathways [37]:

- Farnesoid X receptor (FXR): activated by bile acids, FXR can regulate the expression of genes involved in bile acid synthesis, transport and excretion. It helps reduce BA synthesis in the liver and increases their excretion from the body.
- Takeda G protein-coupled Receptor 5 (TGR5): secondary bile acids, such as LCA and DCA, are potent activators of TGR5, which plays a role in energy metabolism and inflammatory reactions.
- Dysbiosis: imbalances in the gut microbiota can lead to an increase in harmful secondary bile acids, such as DCA and LCA, which have been linked to promoting liver inflammation, damage and carcinogenesis.

These specific microbial interactions with bile acids underscore the complex and crucial role of the gut microbiota in maintaining bile acid homeostasis and influencing the host metabolism. Disruptions in these processes can have profound effects on liver health and the development of diseases such as hepatocellular carcinoma [37].

## 7. Microbiota, Bile Acids and Cholelithiasis

Reports on the adaptive properties of the composition of intestinal microbiota and its metabolites depending on the diet of the host have been well described in previous works [46,47,48]. Similarly, the link between the etiology of cholelithiasis and other factors has been investigated. So far, the factors influencing the risk of gallstones have been described in varying amounts of detail, but they mainly concern microbiota, bile acids and diet. Scientific papers describing the relationship between intestinal microbiota and health, including the function of bile salts, do not describe the issue completely and we still have a lot to discover. The subject of the bile duct microbiota, which plays a huge role in the proper functioning of fat digestive processes, remains incompletely understood. The microbiota of the biliary tract and the gallbladder was analyzed and it was shown that in acute cholecystitis and gallstone patients *Enterobacteriaceae* dominated in the biliary tract [49,50]. The results shown in Wu et al.’s study presented significant differences in gut microbial components between 38 healthy subjects and 29 gallstone patients and showed that the biliary microbiota was mostly composed of the phyla *Bacteroidetes*, *Actinobacteria*, *Proteobacteria* and *Firmicutes* and the genus *Bacteroides* [51]. There was an overgrowth of the bacterial phylum *Proteobacteria*, which generally includes a wide variety of pathogens such as *Escherichia*, *Helicobacter*, *Vibrio* and *Salmonella* in the gut of gallstone patients. These pathogens have been associated with intestinal dysbiosis and gastrointestinal tract diseases. What is more, gut bacterial genera such as *Faecalibacterium*, *Roseburia* and *Lachnospira* were significantly reduced among gallstone patients, and the especially harmful abundance of *F. prausnitzii* has been related to gut dysbiosis, for example, in patients with CD [11,52]. In another study, it was observed that the four mentioned phyla, *Bacteroidetes, Actinobacteria, Proteobacteria* and *Firmicutes,* were dominant in the human gallbladder microbiota of patients suffering from cholelithiasis [53]. What is more, it has been found that *Listeria monocytogenes* or strains of *Salmonella* are able to survive and grow in the gallbladder, which in fact might be a reservoir for these bacteria. Also, one of the few bacteria frequently described as inhabiting the gallbladder is *Salmonella enterica* [54,55]. What is more, as several studies have pointed out, there is a link between cholelithiasis and gallbladder *Helicobacter pylori* infection, and we do have knowledge about the influence of *H. pylori* on the development of gallstone risk in humans [56,57,58,59]. The relative abundance of the family *Propionibacteriaceae* in patients with cholelitiasis was lower than in healthy controls and, in contrast, the abundance of the family *Prevotellaceae, Bacteroidaceae, Porphyromonadaceae*, and *Veillonellaceae* was higher [60].

Cholesterol gallstone formation is mainly described as an effect of the previously mentioned cholesterol accumulation, cholesterol hypersecretion and the balance disturbance of bile components. As the cholesterol content of bile increases, cholesterol becomes supersaturated, causing excess cholesterol to precipitate and accumulate in the gallbladder, which may develop into cholelithiasis [61]. Moreover, the role of mucin overproduction in the formation of cholelithiasis has been described [62]. One of the bile acids, namely ursodeoxycholic acid (UDCA), is considered to reduce the concentration of mucin proteins and thus contribute to reducing the formation of cholesterol crystals in patients with cholelithiasis. For this reason, its use as a pharmacological treatment for gallstones has been proposed [63]. However, in addition to optimistic forecasts of using UDCA as a pharmacological treatment for gallstones, there are also some unfavorable aspects of its use, including the risk of causing cholestasis and malabsorption of bile acids [64]. Endoscopic retrograde cholangiopancreatography (ERCP) is a clinical procedure used in the diagnosis and treatment of cholelithiasis. It is performed for choledocholithiasis with or without cholangitis, the management of pancreatic duct stones, bile and pancreatic leaks and benign and malignant strictures [65]. In the era of greater technological progress in medicine, we have the opportunity to perform specific techniques of this procedure in patients with a surgically altered anatomy, such as selective cannulation with overtube-assisted enteroscopy, EUS-directed transgastric ERCP or laparoscopic surgery assistance. Cholangioscopy and pancreatoscopy are currently important methods of therapy in large bile duct stones and bile duct strictures [65]. On the other hand, the instruments used in ERCP can have a significant impact on post-interventional complications, specifically post-ERCP pancreatic reactions. According to most epidemiological data, the occurrence of post-ERCP pancreatitis rate ranges from 1% to 10%. The retrospective study of Boicean et al. [66] showed that 35.8% (n = 48 out of 134) of patients after ERCP developed post-ERCP pancreatitis at 24–48 h post-intervention. Additionally, most of the patients with post-ERCP complications were female (47.1% vs. 24.2%; *p* = 0.006); however there is no pathophysiological mechanism explanation for this observation. There are a few reports of the role of biliary or pancreatic stent placement in the prevention of pancreatitis by maintaining the patency of the bile duct and preventing the accumulation of gallstones [67]. However, the statements are ambiguous as when the ERCP occurs in the biliary or pancreatic pathway or at the Oddi Sphincter, the surrounding organs undergo papillary edema due to the mechanical, thermal and chemical trauma caused by the intervention. This papillary swelling is also likely to impact the surrounding tissues, which results in blocking normal pancreatic fluid outflow, leading to the development of post-ERCP pancreatitis [66].

In the etiology of cholelithiasis, diet has been considered one of the most important risk factors due to its influence on changes not only in the modification of gallbladder motility, but also in composition of bile salts [68]. According to previously published works, in westernized countries gallstones are mostly (at least 70%) composed of cholesterol, and their origin has links with dyslipidemia, obesity and visceral adiposity, metabolic syndrome and type 2 diabetes as well as insulin resistance, which characterize broad metabolic abnormalities and altered cholesterol homeostasis [68,69,70,71,72,73]. These components have been associated with an elevated occurrence of cholelithiasis and liver steatosis, which have been linked to metabolic syndrome as inherent comorbidities [74,75,76]. A study by Gutiérrez-Díaz et al. involving 28 participants showed that the majority of patients (64.3%) diagnosed with gallstones indicated limiting the consumption of fiber (legumes and vegetables in total), but also dairy products and red meat, which could be related to worse digestive tolerance of these ingredients and the severity of symptoms after consuming them [77]. These patients had a lower consumption of soft drinks and higher consumption of potatoes. This study also showed a lower intake of phenolic compounds, flavonoids, anthocyanins and lignans compared to the control group. Also, the intake of seafood and meats was positively associated with the *Pasteurellaceae* family, and specifically with the genus *Haemophilus*, the intake of dairy products was negatively correlated with the abundance of the family *Bacteroidaceae*, the phylum *Bacteroidetes* and the genus *Bacteroides* and eggs were inversely associated with the abundance of *Proteobacteria* and *Xanthomonadaceae*. Moreover, *Pasteurellaceae* correlated directly with legumes, which were negatively correlated with the relative abundance of this bacterial family [77].

Due to the properties of *Lactobacillus acidophilus* (ATCC 43121) and *Lactobacillus fermentum* (MF27) in reducing the level of lipids, total cholesterol and low-density lipoprotein (LDL) in the serum and liver, it has been suspected that these bacteria may influence mucin biosynthesis and the formation of cholesterol biliary stones. The hypocholesterolemic effect in blood serum and the preventive effect on the development of cholelithiasis of these probiotics were confirmed in a study by Oh et al. on mice induced with a lithogenic diet (LD). The effects of probiotics were due to lowering the HMG-CoA reductase expression in the liver and a reduction in the expression of mucins, including MUC5AC and MUC5B, inside the gallbladder [61]. The authors of this publication concluded that *Lactobacillus* might have a preventive effect against the formation of cholesterol gallstones in the gallbladder and, if taken continuously, can be used clinically to prevent the formation of cholesterol gallstones [61].

## 8. Microbiota and Bile Acid Changes after Bariatric Surgery

The relationship between gut microbiota and bile acids is intriguing and in both directions: bile acids are able to modulate the gut microbiota profile, and vice versa, the pool of bile salts is shaped by bacterial metabolism. In addition, both bile acids and the gut microbiota are modified by bariatric surgery. The composition of the gut microbiota after surgery is affected by changes in diet (often a low-fiber diet), differences in the anatomy and pH of the gastrointestinal tract, altered gastrointestinal transit time and bile acid metabolism [78,79]. The most common changes noted in the study after surgery include a decrease in *Firmicutes* and a concomitant increase in *Bacteroidetes* after SG and *Proteobacteria* after RYGB [80]. Reduced gastric volume after surgery promotes an increase in the pH of the stomach and distal intestinal tract, which in turn promotes the presence of *Akkermansia muciniphila*, *Escherichia coli* and *Bacteroides* spp. [81,82]. In addition, there is an increase in microbial diversity that includes an increase in the abundance of microorganisms belonging to the *Verrucomicrobia* and *Fusobacteria* types, while the proportion of *Actinobacteria* decreases [82]. Based on the systematic review by Davies et al. [80], there is an increase in abundance of the following bacteria after RYGB surgery: *Akkermansia* (*Verrucomicrobia*), *Escherichia* (*Proteobacteria*), *Klebsiella* (*Proteobacteria*), *Roseburia intestinalis* (*Firmicutes*) and *Escherichia coli* (*Proteobacteria*); there is also a decrease in the abundance of the following: *Bifidobacterium* (*Actinobacteria*), *Faecalibacterium prausnitzii* (*Firmicutes*) and *Coprococcus comes* (*Firmicutes*). In turn, an increase in abundance of the following bacteria is recorded after SG: *Bulleidia* (*Firmicutes*), *Roseburia intestinalis* (*Firmicutes*) and *Faecalibacterium prausnitzii* (*Firmicutes*); there is also a decrease in the abundance of *Coprococcus comes* (*Firmicutes*) [80]. Patients who developed gallstones after bariatric surgery had a higher abundance of *Ruminococcus gnavus* (this microbe was previously identified as a biomarker for gallstones) and those who did not develop cholelithiasis had a higher abundance of *Lactobacillaceae* and *Enterobacteriaceae* [83,84]. *Lactobacilli* (an anaerobic bacteria) produce BSH, which deconjugates bile acids in the small intestine and plays a role in bile acid-mediated signaling pathways, which regulate glucose metabolism, lipid absorption and energy homeostasis. *Lactobacillaceae* was also studied as a possible cholesterol-lowering probiotic [85,86,87]. Moreover, increased bile acid concentrations (compared to weight-matched subjects who did not undergo surgery) are noted among those who have undergone RYGB surgery (2–4 years earlier). Bile acid concentrations have been shown to be negatively correlated with postprandial blood glucose levels and GLP-1 secretion (which may suggest a beneficial effect of bile acids on improving glucose metabolism after surgery) [88,89]. In the case of SG, an increase in the pool of circulating serum bile acids was also noted, as were the concentrations of conjugated and unconjugated bile acids regardless of energy restriction [88,89]. It is also worth noting that after bariatric surgery, not only the amount, but also the composition of bile acids changes. A study performed on SG patients demonstrated that after the surgery, the serum level of CA decreased (a 12-α-OH bile acid), and the serum level of taurine-conjugated lithocholic acid (LCA; a non-12-α-OH bile acid) increased [90]. Also, in another study, it was noted that the levels of bile acids other than 12-α-OH increased in both RYGB and SG patients one year after surgery, but the increase was greater in RYGB patients [91]. The amount and type of bile reaching the intestine can alter the gut population due to its antibacterial effect. Studies have shown that a low level of bile salts favors the proliferation of Gram-negative bacteria, while high levels of bile salts favor the proliferation of Gram-positive bacteria [92]. One study [93] reported a higher level of primary and secondary bile acids in SG-operated individuals. The authors concluded that this may be related to the fact that the Gram-negative bacteria *Enterobacteriaceae* were increased in RYGB, while the Gram-positive bacteria *Bifidobacteriaceae* were reduced. As we know, these are two families that have the ability to metabolize bile acids. *Bifidobacteriaceae* are bacteria with a high BSH capacity, while *Enterobacteriaceae* lack this capacity but are able to transform bile acids through dehydroxylation [93,94]. In addition, the main differences observed in the bile acid pool between RYGB and SG were the relatives to the primary bile acid cholate, one of the two major bile salts in the liver together with chenodeoxycholic acid [93]. One of the secondary bile acids—UDCA—significantly reduces the risk of both asymptomatic and symptomatic gallstones after bariatric surgery. A dose of 600 mg/day is associated with improved compliance and better outcomes regardless of type of surgery [95]. Administration of UDCA, tauroursodeoxycholic acid (TUDCA) or glycoursodeoxycholic acid (GUDCA) prevented the loss of the *Clostridium* cluster XIVa and increased the abundance of *Akkermansia muciniphila* [96]. A summary of studies on microbiota and bile acids after bariatric surgery is shown in Table 1.

## 9. Microbiota Modulation and Fecal Microbiota Transplantation in the Context of Gut–Liver Axis

Many studies in recent years have indicated that the gut microbiota plays a key role in maintaining health or developing disease [100]. These microorganisms perform a number of key functions in maintaining the homeostasis of the human body, participating in digestion, production of B and K vitamins, preventing colonization by pathogens and supporting the immune system [101,102]. An extremely important function of the microbiota is the production of short-chain fatty acids, whose beneficial effects on the human body take place on many levels—they have been observed to have beneficial effects on body weight, regulation of hunger and satiety, lipid metabolism, tissue sensitivity to insulin and the ability to strengthen the intestinal barrier and thus reduce inflammation [103]. The intestinal microbiota also has the ability to protect the host from pathogens by, among other things, competing with them for nutrients, producing antimicrobial substances (such as bacteriocins) and stimulating host cells to produce mucus as well as proteins with antimicrobial properties [104]. The intestinal microbiota is unique to each individual, but it is worth remembering that its condition is influenced by many factors. These include type of birth, age, body weight, medications taken, diet, level of physical activity, quality of sleep and stress [105,106]. Due to the fact that the gut microbiota forms a multidirectional axis connecting it to other organs (it works by communicating with nervous, endocrine, humoral, immune and metabolic pathways), disorders in its composition (dysbiosis) can significantly contribute to the development of many diseases and disorders [107]. Among the negative consequences of the development of intestinal dysbiosis is the occurrence of disorders in the gut–brain axis (anxiety, depression and irritable bowel syndrome), gut–brain–endocrine axis (regulatory, metabolic, behavioral and hormonal disorders), gut–heart axis (cardiovascular diseases, atherosclerosis and hypertension), gut–lung axis (chronic obstructive pulmonary disease), gut–pancreas axis (diabetes), gut–bone axis (osteoporosis), gut–muscle axis (muscle impairment and sarcopenia), gut–skin axis (acne, psoriasis and atopic dermatitis), gut–reproductive axis (ovarian cancer and infertility), gut–kidney axis (chronic kidney disease and nephropathy) and gut–bladder axis (urinary tract infection) [107]. However, the gut–liver axis seems to be of greatest importance in the topic of this literature review.

The gut–liver axis is a complex system of interactions between the gut and liver that plays a key role in maintaining the body’s health and balance. The microbiome and liver interact through the portal vein, which transports products from the intestines to the liver and bile and antibodies from the liver to the intestines [108]. The gut–liver axis includes the gut microbiota, whose metabolites can affect liver function (e.g., short-chain fatty acids and lipopolysaccharides), the intestinal barrier (a properly functioning intestinal barrier prevents harmful substances, including pathogens, from entering the bloodstream; damage to the barrier can lead to endotoxemia associated with increased bacterial toxins in the body, which in turn can cause inflammation and liver damage), the immune system (immune cells in the intestine can communicate with the liver, affecting the inflammatory response and regenerative processes in the liver) and bile acids (in the intestine they are converted by intestinal bacteria and reabsorbed into the liver, affecting lipid and glucose metabolism). Abnormalities in the functioning of the gut–liver axis can contribute to the development of a number of diseases, such as inflammatory bowel disease, non-alcoholic fatty liver disease and cirrhosis. Currently, in the scientific literature, we have some observations regarding the transplantation of intestinal microbiota, also in the context of cholangitis and hepatic pathology [108,109].

Patients with hepatic pathology suffer from poor digestion and intestinal disorders due to gut–liver axis dysfunction and fundamentally altered gut microbiomes [110]. For instance, a study of micriobiota composition observed with feces analysis showed that patients with hepatic cirrhosis were identified to have a reduction in the species *Lachnospiraceae* and *Ruminococcaceae* and higher levels of *Enterobacteriaceae*, *Enterococcaceae* and *Staphylococcaceae*, which can be described as gut microbiota dysbiosis [111]. There are several methods of fecal microbiota transplantation (FMT). Among them, we distinguish the upper gastrointestinal tract method (naso–duodenal and naso–jejunal) and the lower gastrointestinal tract method (by enema or colonoscopy up to the cecum) [112]. In the study by Xue et al. [113], FMT improved non-alcoholic fatty liver disease (NAFLD) in patients, but the treatment effect of FMT on lean NAFLD participants was better than that on obese NAFLD patients. This might be conditioned by varied characteristics of the gut microbiota between obese and non-obese NAFLD patients, as the FMT resulted in different responses in the decrease in liver fat accumulation among these two groups. Therefore, abnormal liver metabolism and excess steatosis of this organ in obese bariatric patients, associated with excessive food intake and obesity, leads to further damage of the gut microbiota. These relationships resemble a closed circle, as the intestinal microbiome can be modulated by dietary habits and, mutually, diet and weight loss will improve the composition of the intestinal microbiota, hepatic lipid metabolism and the risk of gallstones. Hu et al. [114] found that in patients with gallstone disease, there is enrichment of *Desulfovibrionales*. Also, fecal transplantation of gut microbiota from gallstone patients to a gallstone-resistant strain of mice can induce gallstone formation. *Desulfovibrionales* is found to be related to an enhanced production of secondary bile acids and increase in bile acid hydrophobicity, which results in intestinal cholesterol absorption. Both H2S, the metabolic product of *Desulfovibrionales*, and the bacteria itself influence bile acid and cholesterol metabolism and thus contribute to the formation of gallstone [114].

## 10. Conclusions

The exact mechanism of gallstone formation after bariatric surgery has not yet been clarified. Taking into account the changes in the composition of the intestinal microbiota and bile acids after bariatric surgery, it seems that this may be an important element affecting the risk of developing gallstones. The higher abundance of *Lactobacillaceae* and *Enterobacteriaceae* in patients after bariatric surgery and without gallstones suggests that this may be one of the protective factors for the occurrence of gallstones. In addition, oral administration of ursodeoxycholic acid may also be an important preventive factor. Undoubtedly, there is a need for further research on this topic.

## Figures and Tables

**Table 1:** Summary of the studies on microbiota and bile acid changes after bariatric surgery with consideration of gallbladder stone development.

Author	Number of Examined Patients	Type of Surgery (n Patients)	Post-Surgical Evaluation	Microbiota Changes after Surgery	Bile Acids Changes after Surgery	Additional Information
[93]	16	RYGB SG	no data	Increased *Enterobacteriaceae* and decreased *Clostridiales* and *Bifidobacteriaceae* after RYGB	There was a reduction in most of the primary bile acids with RYGB. In SG, the primary bile acids seemed to be increased. The secondary bile acids were reduced in RYGB and increased in SG	After RYGB, an increase in *Proteobacteria* and *Veillonella* was observed, the genus *Blautia* from *Clostridiales* decreased in the same way as the family *Bifidobacteriaceae* and its genus *Bifidobacterium*. No significant changes were observed after SG.
[83]	88	RYGB (82) OLGB (6)	2 years	Increased *Bacteroidetes*, *Firmicutes*	Higher concentrations of secondary bile acids were detected in patients with gallstones. In patients without gallstones, the bile acids glycochenodeoxycholate 3-sulfate, glycochenodeoxycholate glucuronide, glycocholate, glycodeoxycholate 3-sulfate, glycohyocholate, glycolithocholate sulfate, taurochenodeoxycholic acid 3-sulfate and taurolithocholate 3-sulfate were increased	Patients who developed gallstones after surgery had a higher abundance of *Bacteroides intestinalis*, *Finegoldia magna*, *Ruminococcus gnavus* and *Prevotella buccalis* and those who did not had a higher abundance of *Lactobacillaceae* and *Enterobacteriaceae.*
[88]	no data	RYGB SG	no data	Increased *Proteobacteria* and *Bacteroidetes*, and decreased *Firmicutes*	Serum bile acid levels are significantly increased 2–4 years after RYGB and both RYGB and SG can result in increased circulating levels of BA	Patients who developed gallstones after RYGB had a higher abundance of *Escherichia coli*, *Klebsiella pneumoniae*, *Veillonella dispar* and *Veillonella parvula* and those who underwent SG had a lower abundance of *Eubacterium rectale*, *Bacteroides vulgatus*, *Bacteroides sp.3_1_40A*, *Coprococcus comes*, *Ruminococcus obeum*, *Dorea longicatena*, *Lachnospiraceae bact.5_1_63FAA* and *Clostridium sp. L2_50.*
[97]	19	RYGB SG MT	1 year	Increased *Proteobacteria* and *Bacteroidetes* after RYGB, increased *Proteobacteria* and decreased *Bacteroidetes* in SG	no data	The abundance of *Firmicutes* was mostly unaffected after both RYGB and SG. The microbiota changes also caused an increase in the *Bacteroides/Firmicutes* ratio in SG patients, and conversely a strong decrease in the RYGB group.
[98]	14	RYGB SG	1 year	Increased *Firmicutes*, *Actinobacteria* and decreased *Bacteroidetes* after RYGB; increased *Bacteroidetes* after SG	no data	In patients with diabetes persisting 1 year after RYGB or SG, there were no phyla level changes. In patients who achieved diabetes remission after RYGB, there were an increase in *Firmicutes, Actinobacietra* and *Bacteroidetes*, and in those who achieved diabetes remission after SG, there wero no phyla level changes.
[99]	47	BIB	1 year	Increased *Lactobacillus* and *Megasphaera* and decreased *Roseburia*	no data	The two major types of bacteria after surgery were *Lactobacillus crispatus* and *Streptococcus* spp. The next major bacterial population found in obese people after surgery is related to *Megasphaera elsdenii*, which is considered the most important rumen lactate-utilizing bacterium.

RYGB—Roux-en-Y gastric bypass; SG—sleeve gastrectomy; OLGB—omega-loop gastric bypass; BAs—bile acids; MT—medical dietary treatment; BIB—Biliointestinal bypass.
